# Extracting and Classifying Drug Discontinuations From Estonian Electronic Health Records: Development and Validation Study

**DOI:** 10.2196/86183

**Published:** 2026-06-17

**Authors:** Hendrik Šuvalov, Nikita Umov, Maria Malk, Markus Haug, Sven Laur, Marek Oja, Sirli Tamm, Sulev Reisberg, Jaak Vilo, Raivo Kolde

**Affiliations:** 1 Institute of Computer Science University of Tartu Tartu Estonia; 2 Institute of Family Medicine and Public Health University of Tartu Tartu Estonia; 3 Software Technology and Applications Competence Center Tartu Estonia

**Keywords:** natural language processing, NLP, large language model, LLM, Estonian, health care data, drug adherence, drug discontinuations, clinical decision support, data mining

## Abstract

**Background:**

Drug adherence is crucial for chronic disease management, yet treatment discontinuation remains common due to factors such as side effects, inefficacy, or cost. These reasons are often recorded only in free-text clinical notes, making large-scale analysis difficult. While large language models (LLMs) can interpret such unstructured data more effectively than traditional natural language processing methods, few studies have systematically categorized reasons for discontinuation or identified whether the decision was initiated by the patient or the clinician, especially in low-resource languages such as Estonian.

**Objective:**

This study aimed to assess the ability of LLMs to extract and classify reasons for drug discontinuation and identify who initiated it using Estonian electronic health records and characterize the observed discontinuation patterns and initiators for statins and antidiabetic medications.

**Methods:**

We combined prescription data with free-text anamneses from a 10% sample of the Estonian population (2012-2019). LLMs (Llama 3.1-70B and GPT-4o) were applied to extract discontinuation phrases and reasons, classify them into a clinician-developed taxonomy, and identify who discontinued the treatment. Performance was evaluated on 100 randomly chosen cases per drug group.

**Results:**

Extraction yielded 625 antidiabetic drug and 233 statin discontinuation cases. Validation confirmed a precision of 0.93 to 0.98 for extracting phrases and 0.95 to 0.96 for extracting reasons. Classification of discontinuation reasons achieved weighted *F*_1_-scores of 0.81 to 0.84, whereas classification of who initiated discontinuation achieved weighted *F*_1_-scores of 0.64 to 0.78. Adverse reactions were the most frequent reason overall, accounting for 70% (163/233) of statin discontinuations and 44.8% (280/625) of antidiabetic drug discontinuations. Regarding antidiabetic drugs, treatment inefficacy and contraindications were more common. Patients more often stopped due to adverse reactions or nonmedical reasons, whereas physicians more often initiated discontinuation for contraindications.

**Conclusions:**

LLMs can accurately extract and classify medication discontinuation reasons and show variable performance in identifying discontinuation initiators in Estonian clinical narratives. Both local and proprietary models showed promising results, enabling scalable analyses that complement structured health records. This demonstrates the potential of LLMs to unlock information from clinical notes, turning this underused electronic health record component into a valuable resource for monitoring treatment patterns and detecting adverse event signals.

## Introduction

Drug adherence and persistence are critical for the management of chronic diseases as they directly affect both patient outcomes and health care costs [1]. Nevertheless, discontinuation of prescribed medications is common [2], driven by factors such as adverse events, lack of perceived efficacy, patient preference, and drug cost [3,4]. While understanding these reasons is essential for improving adherence and informing clinical decision-making, they are rarely standardized or systematically coded in electronic health records (EHRs). When such information is recorded at all, it is typically documented in free-text clinical notes, which are difficult to analyze at scale without manual review [5].

Traditional natural language processing (NLP) approaches, including rule-based methods and classic machine learning, can capture common patterns but often fail to detect the nuanced or less frequent circumstances underlying discontinuation decisions. Large language models (LLMs) offer a promising alternative as they are able to handle semantic complexity and variability in clinical text, making it feasible to classify reasons for discontinuation across thousands of records. Clinical narratives such as patient anamneses (case history), though unstructured and heterogeneous, contain valuable information about patients’ experiences and the clinical rationale behind stopping medications and, thus, represent an important but underused data source.

Prior research on extracting and classifying drug discontinuation reasons has primarily relied on classic NLP methods such as rule-based approaches or machine learning models based on bidirectional encoder representations from transformers architectures [5,6]. More recently, LLMs have shown strong performance on these tasks. For example, LLMs have been applied to study discontinuations in web-based forums [7], which provide valuable patient-reported insights but often lack clinical context. EHRs have been analyzed to identify discontinued medications using LLMs [8], but reasons for discontinuation were not categorized. Related work has also applied NLP and LLM-based approaches to adverse drug event detection in clinical narratives and EHR data, highlighting the potential of these methods for pharmacovigilance and medication safety research [9,10]. More broadly, recent studies have demonstrated the utility of LLMs for extracting, structuring, and classifying information from unstructured clinical narratives and EHR data, with a strong performance [11-14]. Research in lower-resource languages is very scarce, and studies addressing whether discontinuation was initiated by the patient or the clinician are also lacking.

In this study, we used LLMs to examine the discontinuation of antidiabetics and statins among Estonian patients. Specifically, we aimed to (1) identify patients who were prescribed a drug from either category and subsequently stopped purchasing it, (2) extract text segments from anamneses that mention discontinuation or reasons for stopping, (3) determine whether the decision was made by the patient or the physician, and (4) categorize the identified reasons using a clinically developed ontology. By linking structured prescription records with unstructured anamneses, we ensured that our cohort truly reflected patients who discontinued therapy and that negative mentions corresponded to actual discontinuation of treatment. This approach provides systematic insights into the drivers of medication discontinuation in chronic disease management in Estonia and demonstrates the utility of LLMs for pharmacoepidemiological studies in a low-resource language.

## Methods

### Dataset Curation

The dataset used for this study was RITA MAITT, a 10% random sample of the Estonian population (n=150,824) from 2012 to 2019 consisting of claims, prescription, and EHR data [15]. For this study, only unique and nonempty anamneses were used.

From the prescription records, we identified individuals who had purchased drugs classified under the Anatomical Therapeutic Chemical (ATC) groups A10 (antidiabetics) or C10 (lipid-modifying agents). To detect discontinuation, we focused on purchases made in 2012 to 2018 and required a subsequent 1-year observation window without further purchases of the same drug, ensuring that the absence of refills reflected treatment discontinuation. This 1-year window was chosen to align with clinical practice, where patients with chronic conditions are typically reviewed by a physician at least annually, and to ensure the reliability of discontinuation reasons, some of which were self-reported and may be less accurate over longer recall periods. Importantly, patients were considered to have discontinued a medication if they stopped purchasing one specific ATC code drug even if they later initiated another drug within the same class (eg, discontinuation of C10AA05 followed by initiation of C10AA07). This ensured that discontinuation was defined at the level of individual drugs rather than drug classes.

After establishing the discontinuation cohort based on these predefined rules, we extracted free-text clinical notes from the EHRs, specifically anamnesis sections documented within 1 year after the last recorded purchase. Anamneses are particularly informative in this context as they often capture both patient-reported experiences and clinician assessments. Unlike structured fields, anamneses provide the narrative detail necessary to understand the circumstances surrounding treatment discontinuation, making them a valuable source for identifying and classifying underlying reasons.

**Figure 1:**
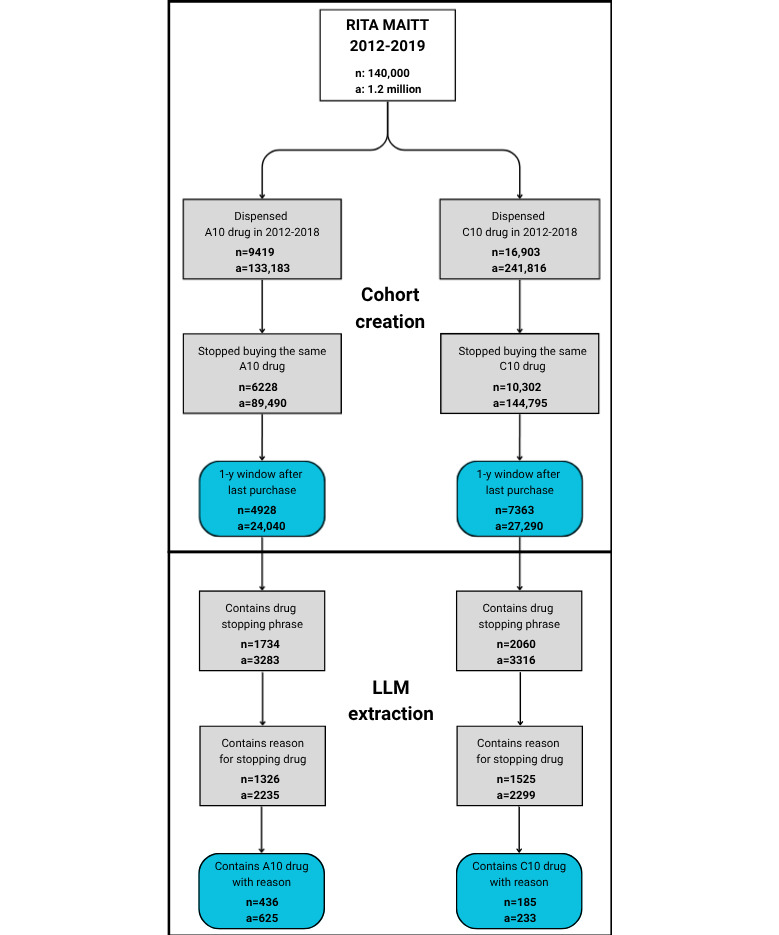
Data flow diagram consisting of two parts: (1) cohort creation—rule-based filtering of anamneses relevant for discontinuation mentions and (2) large language model (LLM) extraction—extracting documents containing discontinuations linked to antidiabetics (A10) or statins (C10). a: anamneses; n: number of patients.

We obtained 24,040 unique anamneses from 4928 patients for A10 and 27,290 unique anamneses from 7363 patients for C10. The data flow diagram is shown in Figure 1 (cohort creation section).

### Extracting Discontinuation Information From Clinical Narratives

On the basis of expert review of the data, many anamneses did not explicitly state discontinuation but, instead, described adverse effects or lack of efficacy. As structured prescription data confirmed that drug dispensing had stopped, such negative experiences (eg, “had no effect” and “caused diarrhea”) were classified as discontinuation reasons even without an explicit mention of stopping.

To systematically identify and structure the information on drug discontinuation from free-text anamneses, we applied an LLM. Specifically, we used Meta-Llama-3.1-70B-Instruct-quantized.w4a16 [16], with a temperature of 0 to ensure consistent outputs across runs. The model was hosted locally in a secure environment to ensure that no sensitive patient data were transmitted or shared externally. Each anamnesis associated with A10 or C10 drug discontinuation in the previous step was provided to the model together with a structured instruction prompt. In a few-shot fashion, each prompt included 10 representative examples curated by a clinician. Although the source data were in Estonian, prompts were written in English as LLMs are predominantly trained on English-language corpora [16,17] and prior work has shown that few-shot prompting with English-language instructions yields the best performance on Estonian EHR data [18]. The prompt template is provided in Multimedia Appendix 1.

To enforce a reproducible and machine-readable format, we constrained the model output using a predefined schema defined using Pydantic, a Python package for validating JSON objects [19]. The schema required the output to include the fields shown in Table 1.

**Table 1:** Extracted fields and their description.

Extracted field	Description
drug_stop_phrase	The full discontinuation-related sentence or clause from the anamnesis (eg, “patient stopped taking metformin because of headaches”), explicitly including the action of stopping, the drug name, and the reported reason
reason_for_stopping	A shorter, normalized span capturing only the reason for discontinuation (eg, “headaches”)
drug_name	The medication name as it appeared in the text

The extracted drug names were subsequently reviewed by a clinician. Any variants, abbreviations, or nonstandard mentions were manually harmonized and mapped to their corresponding ATC codes (A10 or C10). This step ensured alignment between free-text mentions and structured prescription data.

This approach allowed us to capture discontinuation events at the level of individual drugs with both the narrative context (via the *drug_stop_phrase*) and a shorter representation of discontinuation reasons suitable for downstream quantitative analysis. This pipeline is illustrated in Figure 1 (LLM extraction section).

### Classifying Discontinuations

The World Health Organization’s 5-dimension model attributes poor medication adherence to patient, therapy, condition, system, and socioeconomic factors [20]. However, this framework is not specific to discontinuation and is too broad to be applied as practical labeling categories. Subsequent studies have proposed varying taxonomies for discontinuation reasons, often tailored to the type of medication or clinical context [5,7,21]. In this study, we developed a taxonomy of discontinuation reasons based on the framework proposed by Trevena et al [7] but adapted it through a qualitative exploration of our data. Two considerations guided these modifications: first, that the source material reflects physician-entered records rather than patient-reported notes and, second, that the drugs of interest fall within the A10 and C10 classes. The full set of classes and definitions is provided in Table 2.

**Table 2:** Discontinuation reasons and definitions.

Class	Definition
Adverse reactions	Discontinuation due to adverse side effects, allergic reactions, or negative interactions with other medications
Treatment success	Discontinuation due to successful treatment completion or sufficient improvement in health
Treatment inefficacy	Perceived ineffectiveness of the treatment or loss of belief in its efficacy
Contraindication	Discontinuation due to the emergence or discovery of a medical condition or risk factor that makes the continued use of the treatment unsafe or inappropriate
Nonmedical reasons	Discontinuation due to factors unrelated to the patient’s health or the treatment’s medical effects, such as financial constraints, access issues, personal choice, cultural beliefs, or social circumstances
Other	Other medical reasons not explicitly covered by the other categories
Indeterminate	Unclear or unspecified reasons for discontinuation

To assign each discontinuation instance to one of these categories, we again applied an LLM-based approach. All analyses were first performed locally using Meta-Llama-3.1-70B-Instruct-quantized.w4a16. The model was provided with the extracted *reason_for_stopping* text along with the list of possible categories (Table 2) in the system prompt. Each query was framed in a few-shot format using 10 clinician-curated examples to illustrate the mapping process. To ensure structured outputs, we enforced a predefined Pydantic schema, which required a valid JSON response containing the predicted category.

For external validation, the same task was subsequently repeated using GPT-4o (OpenAI) [17] as the extraction of narrowly defined phrases excluded potential privacy concerns. All the extracted reasons were manually reviewed beforehand to confirm that no sensitive information was present. This allowed us to compare outputs between a local deployment and a proprietary model while maintaining patient privacy.

To explore potential differences in discontinuation reason patterns, we stratified texts into switches—texts from patients who switched from one drug to another within the same class—and nonswitches—texts from patients who discontinued without switching—and analyzed the differences. We focused on results from the local model to establish a reproducible workflow for future studies.

In addition, we used the same LLM framework to classify who initiated the discontinuation. The model was prompted (Multimedia Appendix 1) to assign each case to 1 of 3 categories:

- Doctor—discontinuation recommended by the doctor
- Patient—the patient stopped independently
- Unspecified—insufficient information to determine the initiator

### Evaluation Metrics

A random sample of 100 discontinuation cases was evaluated for both statins and antidiabetics. Each case was independently reviewed by 2 medical experts.

These sampled cases were used to evaluate both extraction precision and classification performance. A medical expert assessed whether the extracted discontinuation phrases and reasons were valid representations of the underlying clinical events. Precision was reported as the proportion of outputs judged valid, with 95% CIs calculated using the Wilson method for binomial proportions. For the classification tasks (discontinuation reason and initiator of discontinuation), model predictions were compared against the expert ground truth. Interannotator agreement between the 2 medical experts was assessed using the Cohen κ [22]. We report accuracy (equivalent to microaveraged *F*_1_-scores); weighted *F*_1_-score to account for class imbalance; and the Cohen κ, which adjusts for agreement expected by chance. CIs for classification metrics were estimated using nonparametric bootstrapping with 5000 resamples.

The recall evaluation was concentrated on documents in which no reason for stopping the drug was initially detected. Sets of 100 documents were randomly sampled from this group for both drug classes, and a medical expert reviewed these texts to identify both the reason for stopping the drug and whether the drug of interest was mentioned. The observed number of missed cases was used to estimate the false negative rate for reason detection, and exact binomial CIs were calculated for this rate. The estimated rate and its CIs were then extrapolated to the full set of documents without a detected drug-stopping reason to obtain estimates of the number of false negatives and corresponding CIs. Recall was subsequently calculated, with CIs derived by propagating the false-negative CI bounds through the same formula.

### Ethical Considerations

The medical texts were accessed according to the approval of the Estonian Committee on Bioethics and Human Research (1.1-12/1279), which waived the requirement for informed consent for the use of the data. All data were pseudonymized prior to analysis to prevent the identification of individual patients. All data processing and model inference were performed in a secure local environment to ensure patient confidentiality. The only textual information shared with third-party services consisted of extracted discontinuation reasons that were manually verified to contain no sensitive or personally identifiable information. All other analyses were conducted locally in compliance with applicable data protection regulations and ethical standards governing the use of medical data.

## Results

### Cohort Overview

We focused on clinical texts that simultaneously mentioned a drug from the target group in the extracted phrase and provided an associated reason for discontinuation. Of 24,040 antidiabetic cases, 625 (2.6%) contained both a reason and a drug from the target group, and of 27,290 statin cases, 233 (0.9%) contained both a reason and a drug. For antidiabetic discontinuation, 315 patients had 1 case, 76 had 2 cases, and 45 had 3 or more cases. For statin discontinuation, 152 patients had 1 case, 24 had 2 cases, and 9 had 3 or more cases.

Of the 625 antidiabetic cases, 530 (84.8%) were switches—texts from patients who stopped one drug but started another within the same drug class—and 95 (15.2%) were nonswitches—texts from patients who discontinued without switching to another drug in the same class. Similarly, of the 233 statin cases, 102 (43.8%) were switches and 131 (56.2%) were nonswitches.

The age and sex distributions of patients in the extracted cases were broadly similar to those of all patients who discontinued the respective drugs (Multimedia Appendix 1), suggesting that the extracted subset was reasonably representative of the overall population. However, the relatively high average age of statin and antidiabetic users implies a higher burden of comorbidities, which may introduce additional noise and variability into the extracted discontinuation reasons due to concurrent conditions and treatments. The distribution of time between the last prescription purchase and the epicrisis date containing the analyzed discontinuation case is provided in Multimedia Appendix 1.

Each identified case was further classified by discontinuation reason and by who initiated the decision (patient or physician).

### Evaluation

#### Extraction Performance

Validation of 100 sampled cases per drug group demonstrated a high precision of the extraction process. For antidiabetics, a medical expert reviewed the local LLM-extracted discontinuation phrases and confirmed a precision of 0.93 (93/100, 93% of the cases; 95% CI 0.86-0.97) for discontinuation phrases and 0.95 (95/100, 95% of the cases; 95% CI 0.89-0.98) for extracted reasons. For statins, precision was 0.98 (98/100, 98% of the cases; 95% CI 0.93-0.99) for discontinuation phrases and 0.96 (96/100, 96% of the cases; 95% CI 0.90-0.98) for extracted reasons.

To understand how many cases were missed by the LLM, a negative validation sample of 100 randomly selected discontinuation documents per drug group in which no reason was detected by the model was reviewed. This analysis revealed, for both drug classes, 0 cases in which both a discontinuation reason and the correct drug were present. Consequently, an accurate estimate of the false negative rate and, thus, recall could not be derived for this set of documents.

Using a relaxed criterion requiring only the presence of a discontinuation reason irrespective of drug attribution, this review identified one missed reason among antidiabetic documents and 2 missed reasons among statin documents. On the basis of these observed false negative rates, the estimated recall for reason extraction was 0.91 (95% CI 0.65-1.00) for antidiabetics and 0.82 (95% CI 0.57-0.97) for statins. The calculation of both precision and recall, including their corresponding CIs, is provided in Multimedia Appendix 2.

To highlight the variability in how discontinuation reasons are expressed in the free text, Table 3 summarizes the distribution of unique *reason_for_stopping* patterns by frequency and their overall coverage.

**Table 3:** Distribution of unique discontinuation reason text patterns as they appeared in the texts by frequency and their coverage of the total textsa.

Frequency range	Antidiabetic (A10) patterns and coverage (n=625), n (%)	Statin (C10) patterns and coverage (n=233), n (%)
Exactly once	454 (72.6)	166 (71.2)
2-4 times	58 (22.2)	22 (22.7)
5-9 times	3 (3.2)	2 (6)
≥10 times	1 (1.9)	0 (0)

^a^Coverage represents the percentage of all extracted reason texts accounted for by the occurrence counts of patterns in each frequency range. Patterns were counted after converting the extracted reason text strings to lowercase to avoid treating capitalization variants as distinct patterns.

#### Classification Performance

For discontinuation reasons and initiator (who stopped), predictions from both the local LLM and GPT-4o were compared against annotations from the first medical expert. Table 4 summarizes the performance for both drug groups. One case in antidiabetic reason classification by GPT-4o in the validation set resulted in an error, likely due to a profanity filter, and was considered misclassified. Confusion matrices illustrating true vs predicted labels for discontinuation reason (both models vs the first medical expert) and for discontinuation initiator (local LLM vs the first medical expert) are provided in Multimedia Appendix 1.

**Table 4:** Performance of the local large language model (Llama 3.1-70B; Meta AI) and GPT-4o (OpenAI) in classifying discontinuation reasons and initiators based on validation sets (n=100). Accuracy is equivalent to the micro–F1-score.

Drug group, task, and model				Accuracy (95% CI)		Weighted *F*_1_-score (95% CI)		Cohen κ (95% CI)
**Antidiabetics (A10)**								
	**Reason**							
		Llama 3.1-70B	0.81 (0.72-0.88)		0.82 (0.74-0.89)		0.70 (0.58-0.81)	
		GPT-4o	0.80 (0.71-0.87)		0.84 (0.76-0.90)		0.70 (0.58-0.80)	
	**Initiator**							
		Llama 3.1-70B	0.80 (0.71-0.87)		0.78 (0.68-0.86)		0.69 (0.58-0.80)	
		GPT-4o	—^a^		—		—	
**Statins (C10)**								
	**Reason**							
		Llama 3.1-70B	0.81 (0.72-0.88)		0.82 (0.74-0.89)		0.62 (0.48-0.75)	
		GPT-4o	0.78 (0.69-0.85)		0.81 (0.73-0.88)		0.59 (0.46-0.72)	
	**Initiator**							
		Llama 3.1-70B	0.67 (0.57-0.75)		0.64 (0.54-0.74)		0.45 (0.31-0.59)	
		GPT-4o	—		—		—	

^a^GPT-4o was not evaluated for this task as this would have required submitting longer extracted discontinuation phrases to an external model, which was not performed for privacy reasons.

To contextualize model performance, interannotator agreement between 2 medical experts was assessed for the classification of discontinuation reason and initiator. Agreement was substantial for antidiabetics (Cohen κ=0.71 for reason and 0.69 for initiator) and statins (Cohen κ=0.64 for reason and 0.65 for initiator). These values indicate moderate to substantial consistency between human annotators and provide an upper bound for achievable performance. Model agreement with both medical experts was comparable to interannotator agreement across most tasks, suggesting that model predictions approach human-level reliability for these classification categories.

Corresponding validation results using annotations from the second medical expert are reported in Multimedia Appendix 1.

### Classifying Discontinuations

#### Discontinuation Reasons

**Figure 2:**
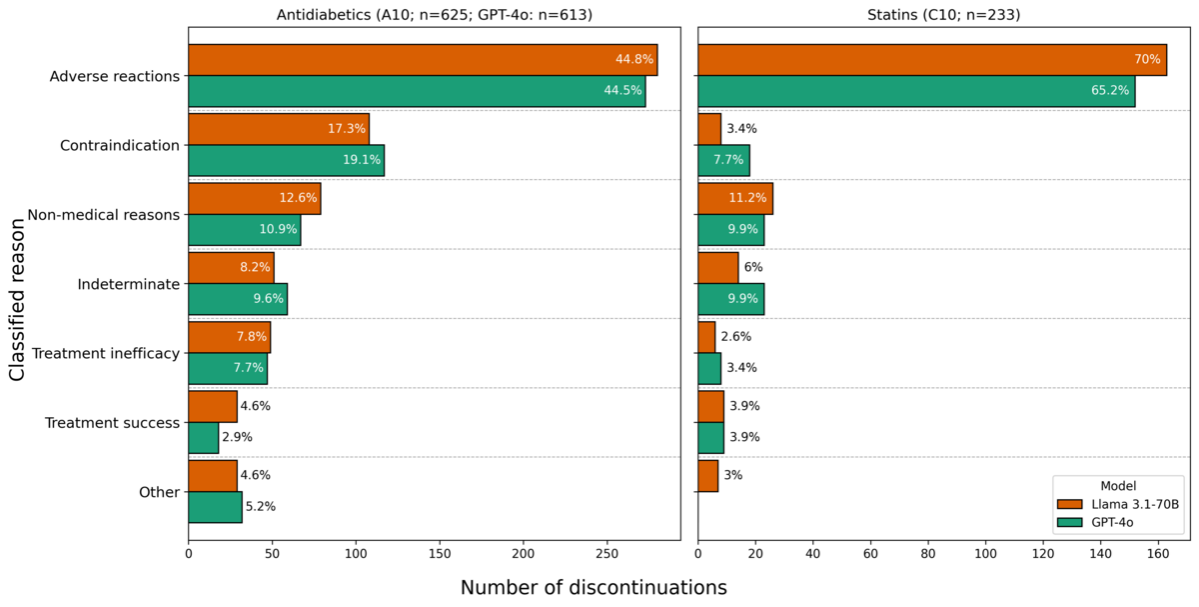
Results of classifying extracted reasons using GPT-4o (OpenAI) and the local Llama 3.1-70B model (Meta AI). For antidiabetics (A10), 12 GPT-4o outputs resulted in an error and were excluded from the figure to reflect unassisted model performance (effective n=613).

Both GPT-4o and the local Llama model were used to classify the extracted reasons for treatment discontinuation (Figure 2). Across most categories, the 2 models produced broadly similar distributions. The main differences were that GPT-4o assigned more cases to *contraindication* and fewer to *other* in the statin group.

When comparing discontinuations of antidiabetic drugs and statins, *adverse reactions* were by far the most common reason in both groups. For statins, using the results from the local LLM, *adverse reactions* accounted for 70% (163/233) of all discontinuations, whereas for antidiabetics, they represented 44.8% (280/625). The lower proportion for antidiabetics can largely be explained by higher frequencies of *contraindication* and *treatment inefficacy* compared with statins.

Instances in which the local LLM classified discontinuation reasons as *adverse reactions* were examined across the full extracted dataset. The corresponding reasons were then grouped into clinically meaningful categories by a medical expert based on domain knowledge without use of a predefined coding schema or standardized taxonomy.

Among antidiabetic drug discontinuations attributed to adverse reactions (280/625, 44.8%), gastrointestinal symptoms were most frequent, accounting for 40.7% (114/280), followed by general malaise at 15% (42/280) and hypoglycemia at 12.9% (36/280). In contrast, among statin-related adverse reactions (163/233, 70%), the leading causes were muscle-related concerns at 32.5% (53/163), general malaise at 20.9% (34/163), and abnormal liver function at 6.7% (11/163).

**Figure 3:**
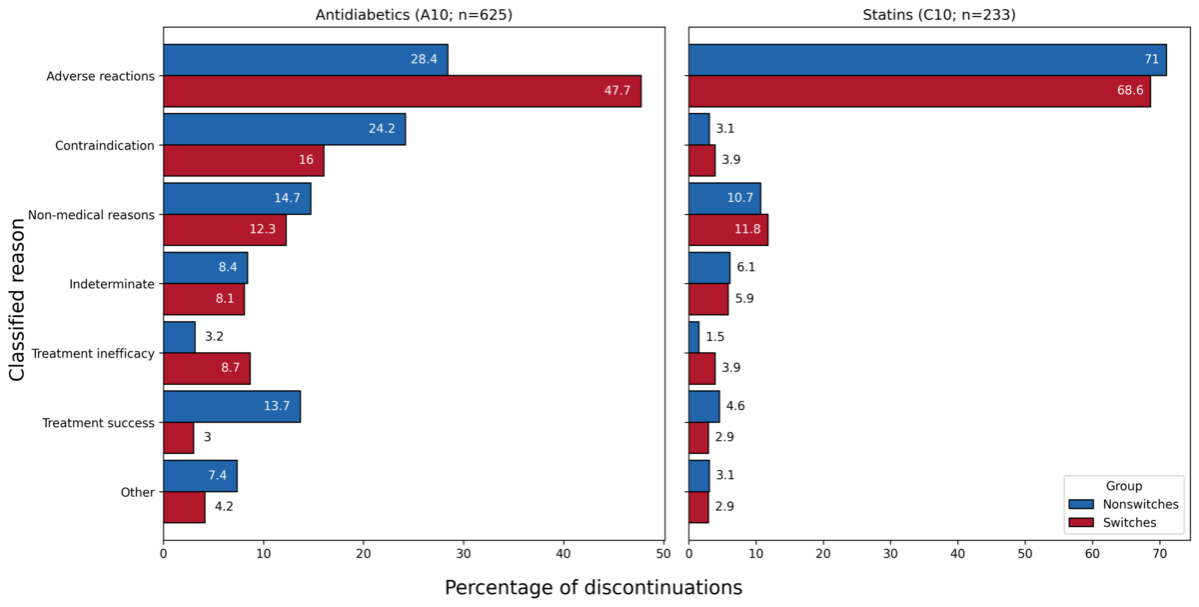
Distribution of discontinuation reasons in patient texts stratified by whether the text came from a patient who switched to another drug within the same class or discontinued entirely using results from the local Llama model.

Using results from the local Llama model, we stratified discontinuation texts based on whether they came from patients who switched to another drug within the same class or discontinued without switching. The results can be observed in Figure 3. Corresponding analyses using shorter gap definitions are provided in Multimedia Appendix 1.

For statins, the distribution of reasons was largely similar between the 2 groups. However, for antidiabetics, differences were apparent: *adverse reactions* and *treatment inefficacy* were more common in texts from switchers, whereas *contraindication* and *treatment success* were more frequent in texts from nonswitchers.

#### Discontinuation Initiator

**Figure 4:**
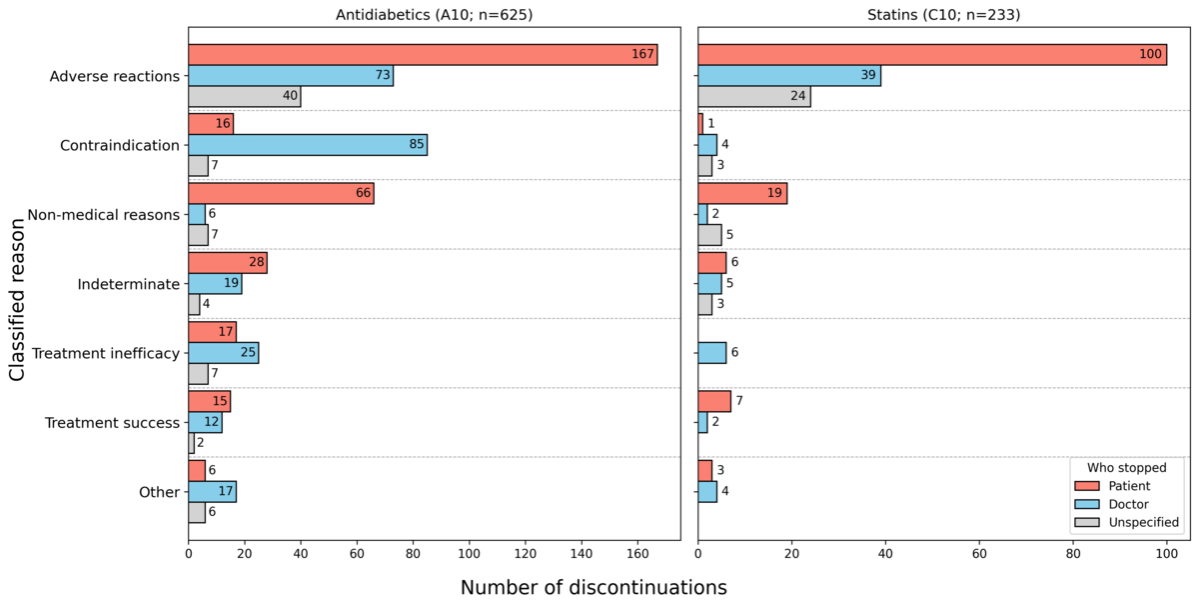
Results of classifying who initiated the discontinuation based on the extracted phrases using the local Llama model.

The local Llama model was used to classify whether the patient or the physician initiated discontinuation. For this task, extracted discontinuation phrases (rather than reasons) were analyzed as they contained the necessary contextual cues. Although both reasons and phrases were derived from the same clinical notes, they differed substantially in length and content. Discontinuation reasons were short (mean 26.8, SD 18.7 characters) and typically consisted of brief, abstract clinical labels (eg, adverse effect and lack of efficacy), whereas discontinuation phrases were considerably longer (mean 77.7, SD 44.4 characters) and frequently included narrative context, references to individuals (eg, patient or clinician actions), and additional clinical details. The results are shown in Figure 4.

Across both drug groups, *adverse reactions* were most often associated with patient-initiated discontinuation. In contrast, *contraindications* were primarily identified as physician initiated. For *nonmedical reasons*, the decision was again more commonly attributed to patients. An interesting difference emerged in the category of *treatment inefficacy*: for antidiabetics, patient-initiated discontinuations were noticeably more frequent than for statins.

## Discussion

### Principal Results

In this study, we showed that LLMs can be used to systematically extract and classify reasons for drug discontinuation from Estonian unstructured clinical narratives. By combining structured prescription data with free-text anamneses, we ensured that discontinuations were real events and that narrative mentions reflected meaningful reasons rather than speculation. This step also allowed us to substantially reduce the number of documents to annotate, focusing on only those we knew were relevant (51,330/1,200,000, 4.3% of the documents), thereby increasing efficiency and minimizing unnecessary review.

Our results show that the local model (Llama 3.1-70B; Meta AI) achieved high precision in extracting the discontinuations. Although recall could not be directly estimated for the final drug-specific extraction due to the absence of observed false negatives in the validation sample, the secondary analysis at the level of reason extraction suggests that the model captures most discontinuation reasons, with only a small number of missed cases observed. For classifying the reason for discontinuation, both the local model and GPT-4o achieved strong results. In the case of classifying the initiator of the discontinuation, the local model performed slightly worse than for reason classification. A possible explanation is that, because the documents are written from the perspective of the physician, it can be challenging to identify the subject of the statement from the context. Moreover, a likely contributing factor is the linguistic and stylistic characteristics of Estonian clinical documentation, which frequently uses implicit or agentless constructions that do not explicitly state who performed the action. For example, expressions such as “*statiinid* - *lõpetatud*” (“statins - stopped”) describe the discontinuation event without specifying whether the decision was made by the physician or the patient. Error analysis showed that these agentless cases accounted for a disproportionate share of misclassifications in the initiator classification task.

The most frequent discontinuation reason was *adverse reactions*, which accounted for a much larger share among statin users. In contrast, for antidiabetic drugs, other drivers such as *treatment inefficacy* and *contraindications* played a more prominent role, likely reflecting challenges in glycemic control and the need to manage comorbidities. Expert review of reported *adverse reactions* indicated that the most common reactions for statins were muscle related, whereas for antidiabetic drugs, they were primarily gastrointestinal symptoms, consistent with previously published literature [23,24].

The observed patterns of switching vs nonswitching provide insights into patient behavior. For antidiabetic drugs, patients were more likely to switch when experiencing *adverse reactions* or perceived *treatment inefficacy*, reflecting the direct and immediate effects on daily glycemic control. For statins, switches were less common, and reasons for switching closely mirrored complete discontinuations. Among antidiabetic nonswitchers, *treatment success* and *contraindications* were more frequent, suggesting that patients are more likely to stop entirely when they feel that the therapy is effective or not suitable, whereas they continue adjusting treatment when efficacy or tolerability is an issue.

Classification of who initiated discontinuation revealed mostly expected patterns, with patients more often stopping due to *adverse reactions* or *nonmedical reasons* and physicians more often initiating discontinuation for *contraindications*. Notably, for antidiabetics, *treatment inefficacy* was much more patient driven than for statins. Analysis of the texts showed that, in these instances, patients measured their blood sugar levels, perceived no effect from the medication, and decided to stop taking it.

### Limitations

First, recall could only be estimated on a limited sample of negative cases. While extracted discontinuation reasons showed high precision, recall was assessed by manually reviewing 100 randomly selected anamneses per drug group in which no reason was detected. This negative validation step identified few missed reasons and provided approximate recall estimates, but it cannot exclude systematic errors such as consistently missing specific categories of discontinuation reasons. Larger negative validation samples would improve the robustness of recall estimation. The results primarily characterize precision and classification fidelity rather than the complete capture of all discontinuations.

Second, causal linkage between symptoms and discontinuation cannot be definitively established from free-text anamneses. Explicit statements such as “the patient discontinued drug X because drug Y caused side effect Z” were uncommon. To mitigate this, discontinuation was confirmed using prescription purchase data, and analyses were restricted to anamneses recorded within 12 months of the last purchase. Nevertheless, some extracted negative mentions may reflect coincidental symptoms rather than true causes of discontinuation.

Third, the number of extracted cases with documented reasons was relatively small, reflecting the rarity of explicit discontinuation reasoning in routine clinical text. This resulted in wide CIs and limited the ability to analyze rare adverse reactions or individual drugs.

Fourth, the validation of discontinuation reason classification for statins showed variability in human annotations, with GPT-4o achieving a substantially lower agreement with the first medical expert (Cohen κ=0.59) than with the second expert (Cohen κ=0.85). This indicates that statin discontinuation reason labels may be subject to interannotator variability, reflecting the inherent ambiguity of clinical text interpretation, which may affect estimates of model performance depending on the reference standard used.

Fifth, the subcategorization of adverse drug reactions into clinically meaningful groups was performed by a single medical expert based on domain knowledge rather than using a predefined standardized taxonomy. This may introduce a degree of subjectivity in the grouping of adverse reactions.

Sixth, although the overall pipeline was designed for scalable analysis, the harmonization of extracted drug names required manual review by a clinician to resolve nonstandard mentions and map them to standardized ATC codes. This step introduces a human bottleneck and currently limits full automation, which may constrain scalability for large-scale deployment without further development of automated normalization methods.

### Future Directions

Future work could enhance this approach in several ways. Performance may improve through evaluation of alternative prompting strategies and newer language models trained on clinical text. The classification scheme could be refined to capture more detailed reasons for medication discontinuation, whereas linking extracted data with longitudinal clinical or genetic information may enable insights into individual susceptibility to adverse reactions. Additionally, applying the pipeline to larger, more diverse EHR datasets could extend its utility for population-level pharmacovigilance and hypothesis generation. Incorporating patient-reported data or other non-EHR sources may further capture discontinuation reasons that are inconsistently documented, such as cost concerns or personal preferences.

### Conclusions

This study demonstrated that LLMs can be applied to Estonian EHRs to identify discontinuation phrases, extract underlying reasons, and classify causes of discontinuation, with lower performance in identifying the initiator of treatment cessation. Validation of 100 sampled cases per drug group showed that, for antidiabetics, precision was 0.93 for extracted discontinuation phrases and 0.95 for extracted reasons, whereas for statins, precision was 0.98 for phrases and 0.96 for reasons. Classification of discontinuation reasons achieved a weighted *F*_1_-score above 0.80 for both antidiabetics and statins, whereas classification of the initiator achieved weighted *F*_1_-scores of 0.78 for antidiabetics and 0.64 for statins. Our findings showed that *adverse reactions* dominated among statin users, whereas *contraindications* and *treatment inefficacy* were more frequent for antidiabetic drugs, with patients and physicians contributing to discontinuation in distinct and clinically consistent ways.

Together, these results highlight the feasibility of transforming this previously underused data source into a valuable asset for pharmacovigilance and routine adverse event signal screening. This methodology is primarily intended for retrospective, population-level surveillance using large real-world datasets that include substantial unstructured clinical text. It enables identification of patterns and potential drivers of medication discontinuation that cannot be detected from structured data. In addition, the approach can support hypothesis generation for adverse event signals or adherence studies. While not designed for real-time clinical decision support, the pipeline demonstrates how LLMs can extract valuable information from narrative low-resource–language EHR data for medication safety surveillance beyond traditionally accessible data sources.

## Data Availability

The medical texts analyzed during this study were used according to the Estonian Committee on Bioethics and Human Research approval (1.1-12/1279). These data are not publicly available because they may contain identifiable information about the participants. Access to the medical texts cannot be granted by the authors and is subject to approval by the relevant ethics committee and applicable data protection requirements. The code for the pipeline is publicly available in GitHub [26].

## Appendix

Multimedia Appendix 1 Prompt templates, cohort comparisons, temporal analyses, classification confusion matrices, secondary validation results, and sensitivity analyses.

Multimedia Appendix 2 Calculations of precision and recall.

## Abbreviations

ATC: Anatomical Therapeutic Chemical
EHR: electronic health record
LLM: large language model
NLP: natural language processing
