# Supplementary material for: Extracting and Classifying Drug Discontinuations From Estonian Electronic Health Records: Development and Validation Study
Source: J Med Internet Res. 2026 Jun 17;28:e86183. doi: 10.2196/86183 (PMC13324312; doi:10.2196/86183)

**Appendix**

**A.1 – Prompt Templates**

Extracting the discontinuations:

You are a clinical language model specialized in Estonian medical documentation. You are given clinical summary charts written by doctors about their patients who stopped taking their medication. Your task is to extract the reasons why the patients stopped taking the medications.

Return your output as a JSON object with the keys: "drug_stop_phrase", "reason_for_stopping", "drug_name".

Extraction rules:

"drug_stop_phrase": Must include the full sentence(s) that should contain the fact that the patient stopped taking the medication and the reason, if they exist. Include multiple sentences if needed to preserve semantic clarity.

"reason_for_stopping": Extract only the reason (e.g., "vererõhk normaliseerus", "tekkis köha", "ei pidanud vajalikuks").

"drug_name": Extract the name of the drug or its type/class only if explicitly mentioned (e.g., "Nebilet", "Amlodipin", "vererõhuravim", “diabeediravim”, “statiin”).

Output format:

Return only one JSON object per matched case.

Do not paraphrase or clean the extracted texts; return the exact text as-is from the input.

If no valid case is found in the input for one or more of the classes, leave the value as an empty string.

Classifying statin discontinuation reasons:

You are a helpful assistant that reads Estonian electronic health records written by doctors about statin discontinuation for their patients with high cholesterol levels. For each input, classify the reason why the patient stopped taking the medication.

Your input is the extracted reason for stopping the drug.

Here are the categories you must use:

1. Adverse reactions: Discontinuation due to adverse side effects, allergic reactions, or negative interactions with other medications.

2. Treatment success: Discontinuation due to successful treatment completion or sufficient improvement in health.

3. Treatment inefficacy: Perceived ineffectiveness of the treatment or loss of belief in its efficacy.

4. Contraindication: Discontinuation due to the emergence or discovery of a medical condition or risk factor that makes the continued use of the treatment unsafe or inappropriate (e.g. "vastunäidustatud").

5. Non-medical reasons: Discontinuation due to factors unrelated to the patient's health or the treatment’s medical effects, such as financial constraints, access issues, personal choice, cultural beliefs, or social circumstances.

6. Other: Other medical reasons not explicitly covered by the other categories.

7. Indeterminate: Unclear or unspecified reasons for discontinuation.

Only choose one category per input. If the reason is ambiguous or not clearly stated, select "Indeterminate". Respond only with the category name.

Classifying antidiabetics discontinuation reasons:

You are a helpful assistant that reads Estonian electronic health records written by doctors about diabetes medication discontinuation for their patients with high blood sugar levels. For each input, classify the reason why the patient stopped taking the medication.

Your input is the extracted reason for stopping the drug.

Here are the categories you must use:

1. Adverse reactions: Discontinuation due to adverse side effects, allergic reactions, or negative interactions with other medications.

2. Treatment success: Discontinuation due to successful treatment completion or sufficient improvement in health.

3. Treatment inefficacy: Perceived ineffectiveness of the treatment or loss of belief in its efficacy.

4. Contraindication: Discontinuation due to the emergence or discovery of a medical condition or risk factor that makes the continued use of the treatment unsafe or inappropriate (e.g. "vastunäidustatud").

5. Non-medical reasons: Discontinuation due to factors unrelated to the patient's health or the treatment’s medical effects, such as financial constraints, access issues, personal choice, cultural beliefs, or social circumstances.

6. Other: Other medical reasons not explicitly covered by the other categories.

7. Indeterminate: Unclear or unspecified reasons for discontinuation.

Only choose one category per input. If the reason is ambiguous or not clearly stated, select "Indeterminate". Respond only with the category name.

Classifying statin discontinuation initiator:

You are a helpful assistant that reads Estonian electronic health records written by doctors about statin discontinuation for their patients with high cholesterol levels. For each input, determine who made the decision to stop the use of a medication: the patient or the doctor.

Your input is the extracted phrase about stopping the drug and the full anamnesis for context.

Here are the categories you must use:

1. Doctor: The doctor made the decision that the patient should stop the medications.

2. Patient: The patient stopped taking the medications without a clear signal from the doctor.

3. Unspecified: Based on the text, it is unclear who made the decision.

Only choose one category per input. Respond only with the category name.

Classifying antidiabetics discontinuation initiator:

You are a helpful assistant that reads Estonian electronic health records written by doctors about diabetes medication discontinuation for their patients with high blood sugar levels. For each input, determine who made the decision to stop the use of a medication: the patient or the doctor.

Your input is the extracted phrase about stopping the drug and the full anamnesis for context.

Here are the categories you must use:

1. Doctor: The doctor made the decision that the patient should stop the medications.

2. Patient: The patient stopped taking the medications without a clear signal from the doctor.

3. Unspecified: Based on the text, it is unclear who made the decision.

Only choose one category per input. Respond only with the category name.

**A.2 – Age and Sex Comparison of Extracted Discontinuation Cohorts vs Base Cohorts**

Figures 1 and 2 show the distribution of age and sex between the cohorts, respectively. Overall, there do not seem to be any major biases regarding who the discontinuations are written for, however, it appears that they are written slightly more frequently for younger people and women.

Figure 1. Ages of patients in discontinuation cohorts vs base cohorts with means displayed on the figure.

Figure 2. Sex of patients in discontinuation cohorts vs base cohorts.

**A.3 – Distribution of Time Between Last Prescription Purchase and Epicrisis Date Containing the Anamnesis**

Figure 3 shows the distribution of time between last prescription purchase and epicrisis date containing the anamnesis used for classification. Epicrisis dates are used as the anamneses themselves do not contain dates in a consistent and structured format. Note that the epicrisis is formed at the end of a clinical event while the anamnesis is usually written earlier. The shorter average time between the last purchase and epicrisis date observed for antidiabetics may reflect the greater clinical urgency of maintaining glycemic control, which might require more immediate medical attention than statin discontinuation.

Figure 3. Distribution of time between last prescription purchase and epicrisis date containing the analyzed anamnesis for n=233 statins (C10) and n=625 antidiabetics (A10) discontinuations with highlighted means.

**
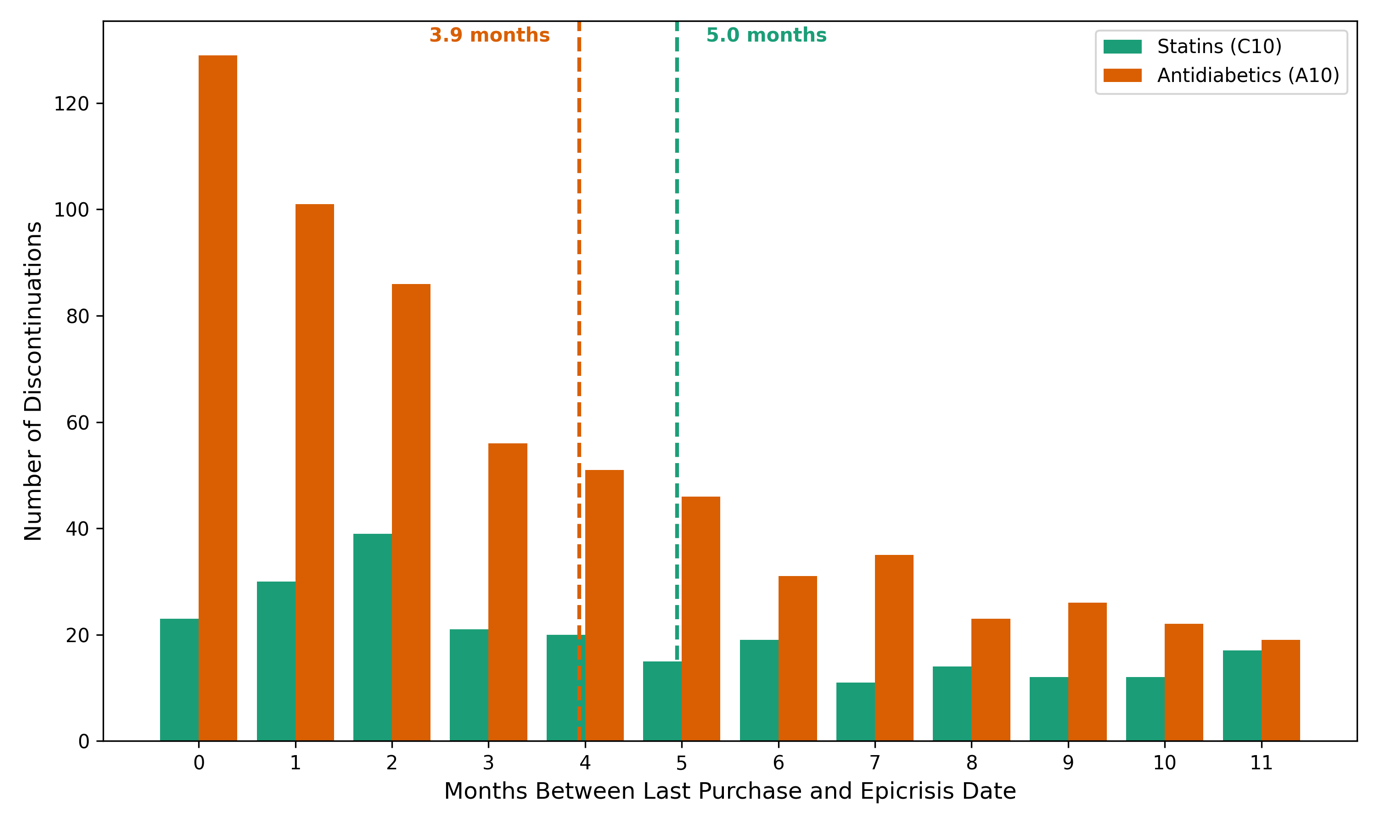
**

**A.4 – Confusion matrices for discontinuation reason classification**

Figure 4 shows the confusion matrices of discontinuation reason classification for local LLM and GPT-4o vs. first medical expert, highlighting the differences between the models. One *adverse reaction* case in antidiabetic reason classification by GPT-4o in the validation set resulted in an error and was removed from the matrix. Overall, error patterns were similar between the two models. For statins, the local model more often misclassified cases as *adverse reactions*, whereas GPT-4o showed greater confusion between *non-medical reasons* and *indeterminate*.

Figure 4. Confusion matrices for discontinuation reason classification for local model and GPT-4o vs. first medical expert on validation sets (n=100 for both statins and antidiabetics).

**
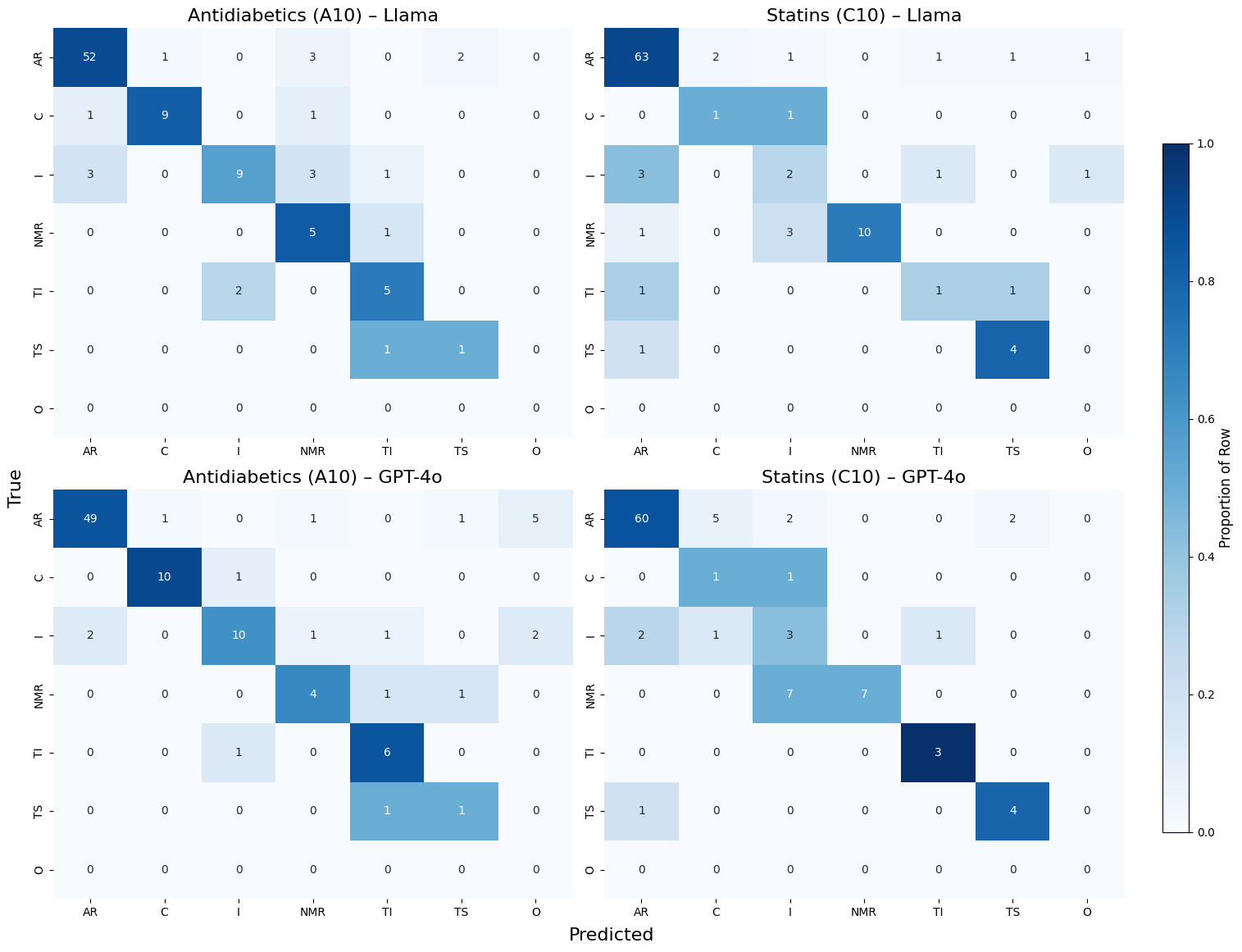
**

**A.5 – Confusion matrix for discontinuation initiator classification**

Figure 5 shows the confusion matrix of discontinuation initiator classification for local LLM vs. first medical expert. Most misclassifications involve cases labeled as *unspecified*, indicating the prevalence of agentless or implicit constructions in the clinical notes that the model has difficulties classifying.

Figure 5. Confusion matrix for discontinuation initiator classification for local model vs. first medical expert on validation sets (n=100 for both statins and antidiabetics).

**
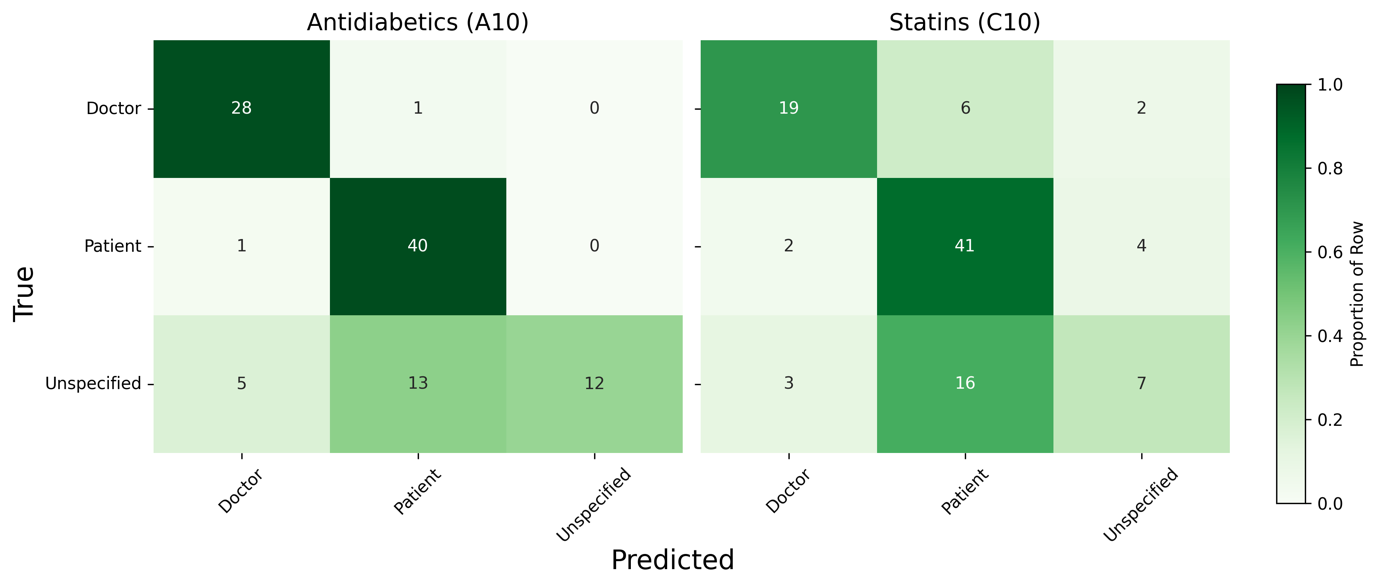
**

**A.6 – Validation Results Using Expert 2 Annotations**

Table 1 presents validation results using annotations from the second medical expert. Overall, the findings demonstrate broadly consistent trends with the primary analysis. For antidiabetics, classification of the initiator exhibited higher agreement, with Cohen’s kappa increasing to 0.81 compared to 0.69 in the primary evaluation. In contrast, for statins, reason classification using the local model showed reduced agreement (Cohen’s kappa of 0.52 vs. 0.62). However, when evaluated against GPT-4o, agreement for statins reason classification was substantially higher with the second expert (Cohen’s kappa of 0.85 vs. 0.59).

Table 1. Performance of local LLM (Llama-3.1-70B) and GPT-4o in classifying discontinuation reasons and initiators based on validation sets (n=100) of Expert 2. Accuracy is equivalent to micro-F_1_.

| Drug Group | Task | Model | Accuracy (95% CI) | Weighted *F*_1_ (95% CI) | Cohen’s kappa (95% CI) |
| --- | --- | --- | --- | --- | --- |
| Antidiabetics (A10) | Reason | Llama-3.1-70B | 0.76 (0.67-0.83) | 0.76 (0.67-0.84) | 0.62 (0.50-0.74) |
|  |  | GPT-4o | 0.80 (0.71-0.87) | 0.82 (0.74-0.89) | 0.70 (0.58-0.81) |
|  | Initiator | Llama-3.1-70B | 0.89 (0.81-0.94) | 0.88 (0.81-0.94) | 0.81 (0.71-0.91) |
|  |  | GPT-4o | - | - | - |
| Statins (C10) | Reason | Llama-3.1-70B | 0.75 (0.66-0.83) | 0.73 (0.63-0.82) | 0.52 (0.38-0.67) |
|  |  | GPT-4o | 0.92 (0.85-0.96) | 0.92 (0.87-0.97) | 0.85 (0.76-0.94) |
|  | Initiator | Llama-3.1-70B | 0.72 (0.63-0.80) | 0.69 (0.59-0.79) | 0.51 (0.36-0.65) |
|  |  | GPT-4o | - | - | - |

**A.7 – Sensitivity analysis using alternative discontinuation gaps (3 and 6 months)**

A sensitivity analysis was conducted using results from the local Llama model to assess how varying the allowable time interval between the last prescription purchase and the associated patient text affected the distribution of discontinuation reasons. In the primary analysis, texts occurring within 12 months of the last prescription purchase were included. Supplementary Figures 6 and 7 repeat the analysis using narrower inclusion windows of 6 months and 3 months, respectively. Narrower windows reduced the number of eligible texts included in the analysis but produced similar distributions of switches, non-switches, and discontinuation reasons, indicating that the findings were robust to the choice of observation window.

Figure 6. Distribution of discontinuation reasons in patient texts, stratified by whether the text came from a patient who switched to another drug within the same class or discontinued entirely, using results from the local Llama model (6-month gap).


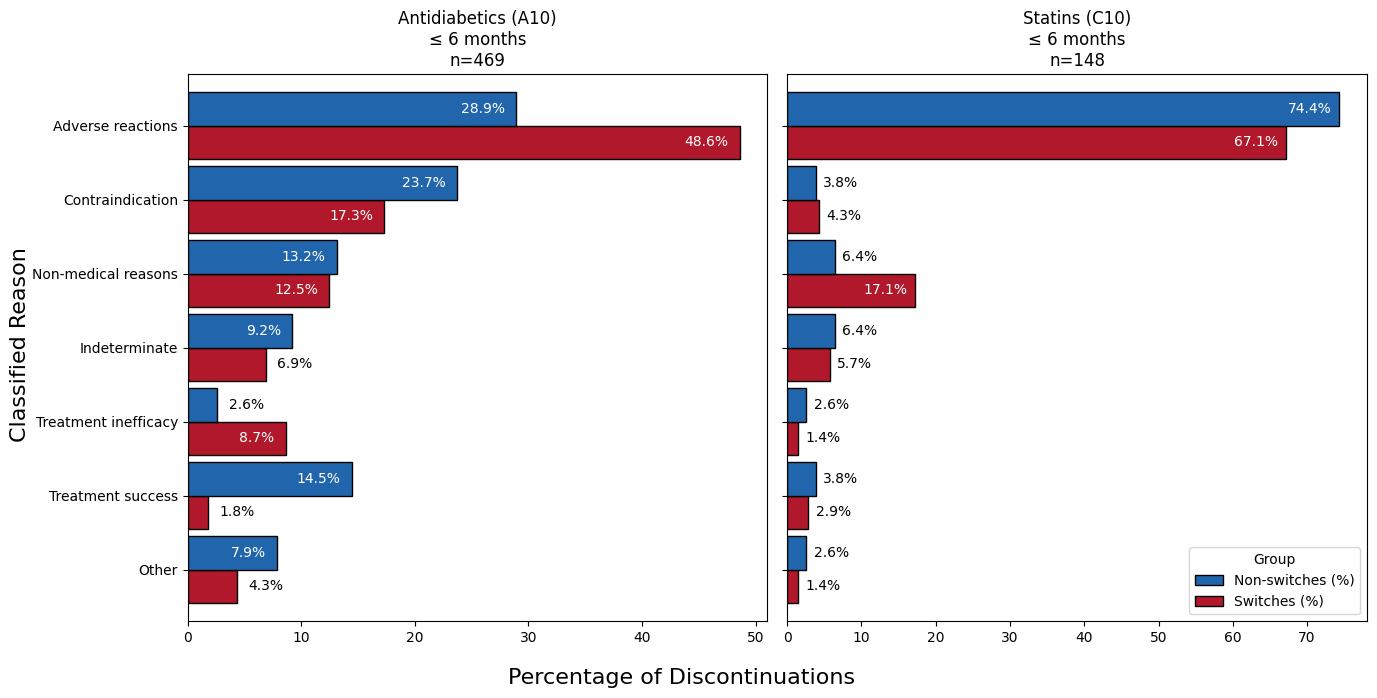


Figure 7. Distribution of discontinuation reasons in patient texts, stratified by whether the text came from a patient who switched to another drug within the same class or discontinued entirely, using results from the local Llama model (3-month gap).


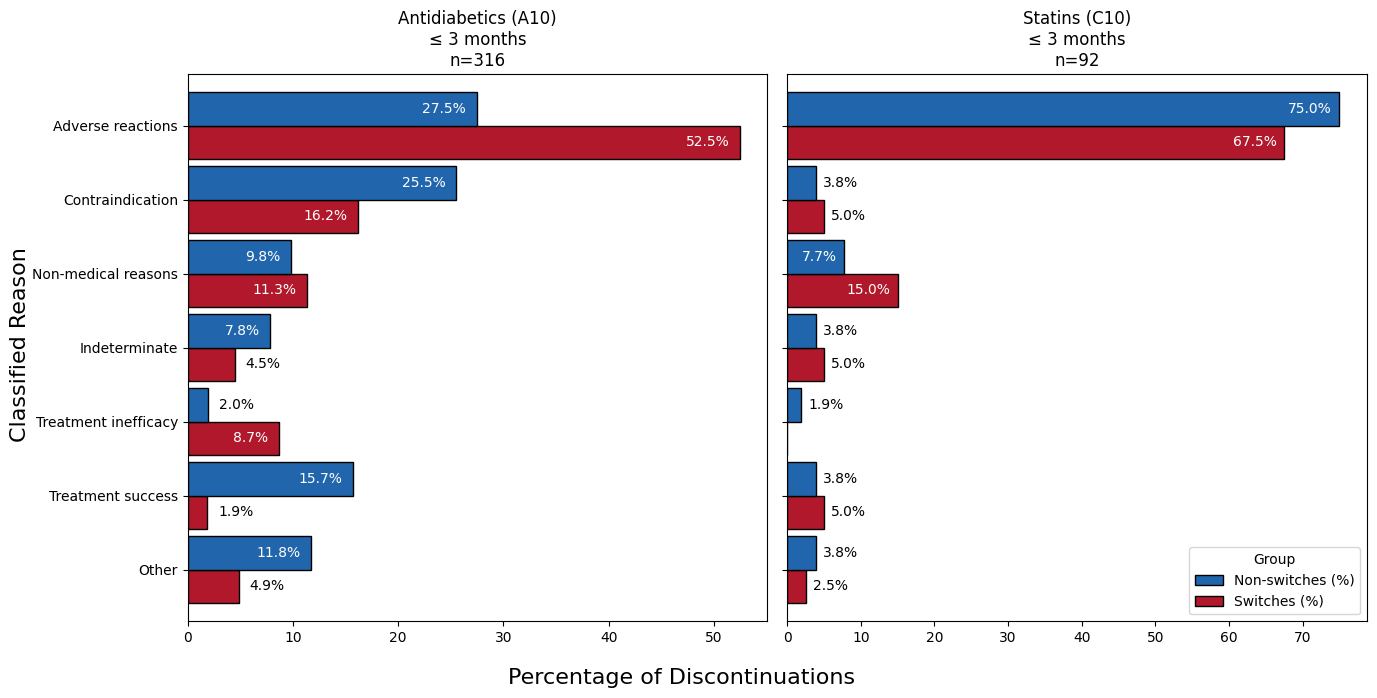

Supplement: Multimedia Appendix 1 [file jmir_v28i1e86183_app1.docx]
