# Supplementary material for: Extracting and Classifying Drug Discontinuations From Estonian Electronic Health Records: Development and Validation Study
Source: J Med Internet Res. 2026 Jun 17;28:e86183. doi: 10.2196/86183 (PMC13324312; doi:10.2196/86183)
Supplement: Multimedia Appendix 2 [file jmir_v28i1e86183_app2.docx]

Supplementary Materials B: Calculation of Precision and Recall

This supplement shows the formulas and numerical calculations used for the precision and recall estimates.

# 1. Precision (Wilson confidence intervals)

Precision was evaluated on a positive validation sample of 100 cases per drug group drawn from the final set of model-identified discontinuation events (625 for antidiabetics and 233 for statins).

p̂ = x / n

Antidiabetics – Discontinuation phrases:

x = 93, n = 100, p̂ = 0.93

Wilson 95% CI = [0.8625, 0.9657] ≈ [0.86, 0.97]

Antidiabetics – Reasons:

x = 95, n = 100, p̂ = 0.95

Wilson 95% CI = [0.8882, 0.9785] ≈ [0.89, 0.98]

Statins – Discontinuation phrases:

x = 98, n = 100, p̂ = 0.98

Wilson 95% CI = [0.9300, 0.9945] ≈ [0.93, 0.99]

Statins – Reasons:

x = 96, n = 100, p̂ = 0.96

Wilson 95% CI = [0.9016, 0.9843] ≈ [0.90, 0.98]

# 2. Recall (negative validation)

Negative validation sample: 100 cases per drug group drawn from model-negative pool (no reason detected, 24040 – 2235 for antidiabetics and 27290 – 2299 for statins). Let p̂_FN denote the observed false-negative rate in the sample and FN̂ denote the estimated number of false negatives in the full model-negative pool.

FN̂ = p̂_FN × N_neg

Recall = TP / (TP + FN̂)

Antidiabetics:

N_neg = 24040 - 2235 = 21805

TP = 2235, p̂_FN = 1/100 = 0.01

Exact 95% CI for p̂_FN = [0.000253, 0.054459]

FN̂ = 0.01 × 21805 = 218.05

Recall = 2235 / (2235 + 218.05) = 0.91

Lower recall bound = 2235 / (2235 + 21805 × 0.054459) = 0.6530
Upper recall bound = 2235 / (2235 + 21805 × 0.000253) = 0.9975

95% CI ≈ [0.65, 1.00]

Statins:

N_neg = 27290 - 2299 = 24991

TP = 2299, p̂_FN = 2/100 = 0.02

Exact 95% CI for p̂_FN = [0.002431, 0.070384]

FN̂ = 0.02 × 24991 = 499.82

Recall = 2299 / (2299 + 499.82) = 0.82

Lower recall bound = 2299 / (2299 + 24991 × 0.070384) = 0.5665
Upper recall bound = 2299 / (2299 + 24991 × 0.002431) = 0.9743

95% CI ≈ [0.57, 0.97]
